# Acute Thyroid Storm Following Thymectomy: A Surprising Result of Undiagnosed Graves’ Disease

**DOI:** 10.7759/cureus.3239

**Published:** 2018-08-31

**Authors:** Mohammadali M Shoja, Omar Nunez Lopez, Ikenna Okereke

**Affiliations:** 1 Surgery, University of Texas Medical Branch, Galveston, USA

**Keywords:** thyroid storm, mediastinum, thymic mass

## Abstract

Postoperative thyroid storm represents a diagnostic dilemma in patients with overlooked hyperthyroid state undergoing a nonthyroid surgery. We report a 30-year-old female with a history of mixed connective tissue disease who presented with an anterior mediastinal mass and underwent a thoracoscopic resection of the mass. On postoperative day 1, she had an acute change in mental status with fever, tachycardia and hypercapnic respiratory failure requiring intubation and mechanical ventilation. An elevated free thyroxine concentration and almost undetectable serum thyroid stimulating hormone suggested thyroid storm as the culprit. The patient was rendered euthyroid after initiation of therapy with propylthiouracil/methimazole, potassium iodide oral solution and systemic steroid. Histopathology of the resected anterior mediastinal mass showed thymic hyperplasia. In retrospect, the patient had hyperthyroid symptoms before surgery, but this diagnosis was overlooked.

Non-thyroid surgeries can trigger thyroid storm in the setting of poorly controlled or overlooked hyperthyroidism. Although uncommon, thyroid storm should be considered in differential diagnosis of perioperative tachycardia and respiratory failure. We emphasize on the importance of preoperative thyroid workup in patients with tachycardia, palpitation, labile blood pressure, unexplained weight changes or poorly controlled anxiety. The significance of a proper preoperative assessment cannot be overestimated.

## Introduction

Thyroid storm (TS) is a life-threatening but treatable endocrine emergency with an inpatient incidence of 0.2 per 100,000 population per year and a mortality rate as high as 40% [1]. Any systemic insult such as infection, severe psychological stress, pregnancy, delivery, myocardial infarction, trauma or surgery and certain medications such as iodinated contrast agents and anesthetic agents can precipitate a TS in predisposed patients. Thyroid storm almost always occurs in the setting of an underlying hyperthyroid state, either overt or subclinical. Untreated thyroid storm can result in fatal cardiac and respiratory decompensation. Therefore, early identification and aggressive treatment are crucial to ensure survival. Mortality secondary to delays in diagnosis and treatment of TS in a patient with overlooked hyperthyroidism sporadically presents in the literature [2-7].

Perioperative TS represents a diagnostic dilemma in patients with an overlooked hyperthyroid state undergoing non-thyroid surgery [8-9]. Manifesting with an acute onset of tachycardia, respiratory failure and a hyperdynamic state, TS can be misdiagnosed as more common perioperative adverse events such as pulmonary thromboembolism or sepsis. We describe our experience with a patient in whom postoperative TS caused respiratory failure and a hyperdynamic circulation that masqueraded as pulmonary thromboembolism. A verbal informed consent was obtained from the patient for publication of this report.

## Case presentation

A 30-year-old African-American woman presented to our institution with a six-day history of progressively worsening neck and anterior upper chest pain radiating to the arms. The pain was excruciating, sharp and constant in nature without any alleviating or aggravating factors. She also had fever, night sweats, progressive fatigue, and shortness of breath. She had experienced an ‘intentional’ weight loss (10 lbs) with diet and exercise. Her exercise tolerance had progressively diminished from walking six miles to barely being able to walk to the bathroom without dyspnea. Her past medical history was remarkable for mixed connective tissue disease (MCTD), fibromyalgia and chronic pain syndrome. Her autoimmune condition was accompanied with elevated serum titers of rheumatoid factor, anti-Ro/Sjogren's syndrome-related antigen A (SSA), anti-cyclic citrullinated peptide, anti-ribonucleoprotein and antinuclear antibodies. Clinically, this manifested with mixed features of rheumatoid arthritis and systemic lupus erythematous over a span of 10 years. As part of her autoimmune workup, she had been tested for thyroid diseases approximately two years previously. Her previous thyroid stimulating hormone (TSH) levels had ranged from 0.56 to 0.77 international microunits/milliliter (uIU/mL) respectively (normal, 0.45–4.70 uIU/mL). She denied smoking cigarette and consuming alcohol.

**Figure 1:**
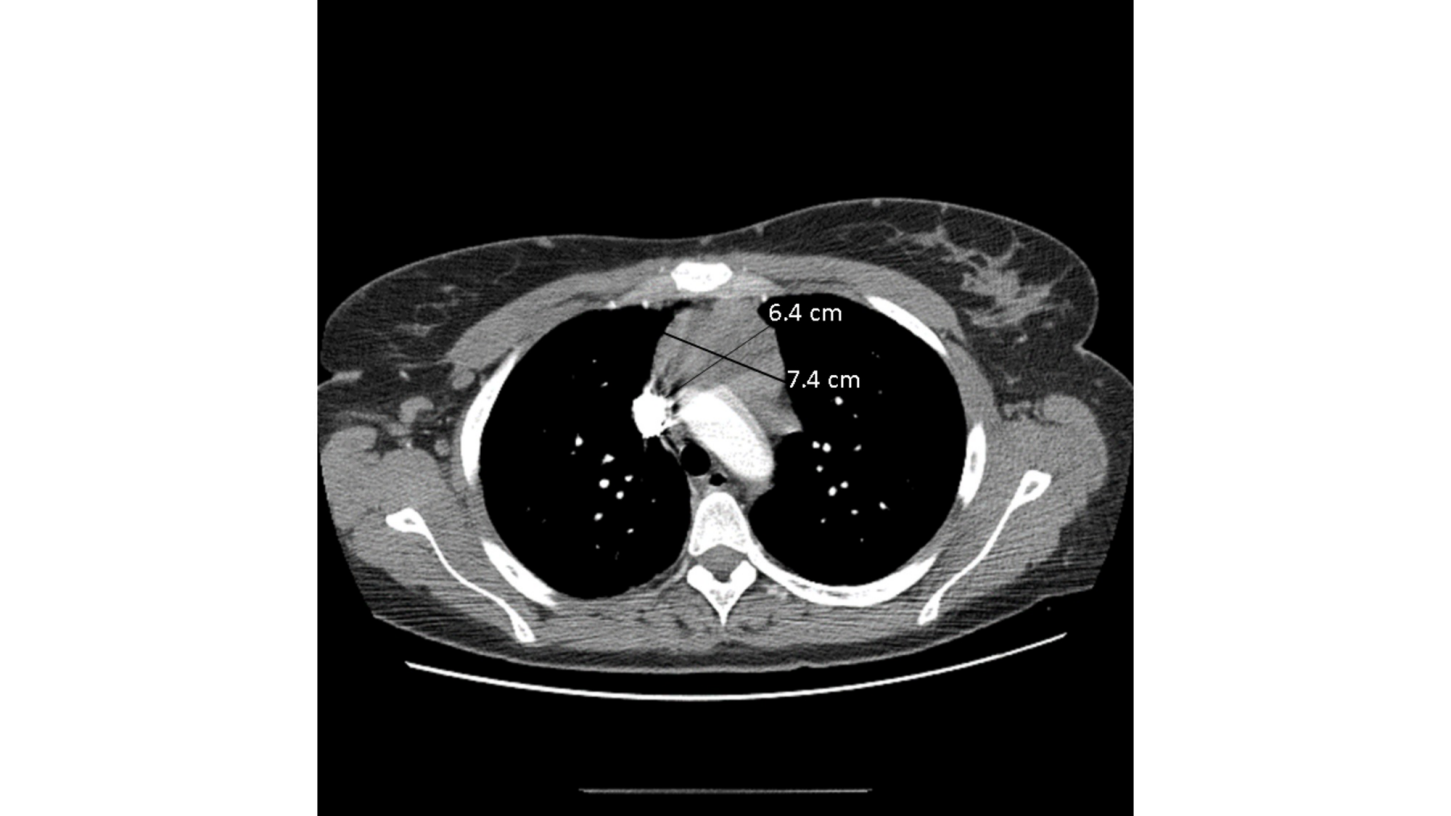
Contrasted computed tomography (CT) images of the chest showing preoperative lobular mass in the anterior mediastinum.

A computed tomography (CT) of the chest with contrast performed to evaluate her shortness of breath revealed a lobular mass in the anterior mediastinum measuring 4.1 x 7.4 x 6.4 centimeters (cm), as represented in Figure 1. Her lower neck and thyroid gland were unremarkable. A percutaneous biopsy was non-diagnostic. She was discharged after recovering from her acute condition and scheduled for elective surgery. Three weeks later she underwent a thoracoscopic resection of the mass and total thymectomy. To perform the surgery, two 1 cm port sites were created. The mass was able to be dissected off the surrounding intra-thoracic structures using an ultrasonic energy device. The mass did not invade the pericardium or any other intra-thoracic structures.

On postoperative day 1, she developed fever to 38 degrees Celsius, tachycardia (150 beats per min) and tachypnea (30 breaths per min). She was agitated and tremulous. A chest CT was negative for pulmonary embolism. Over the next few hours, her condition worsened and she required intubation and mechanical ventilation. An arterial blood gas revealed an evolving mixed metabolic and respiratory acidosis (Table 1).

**Table 1:** Postoperative arterial blood gas results.

	Postoperative day 1	Postoperative day 2 Morning	Postoperative day 2 Evening
pH	7.40	7.28	7.34
Partial pressure of carbon dioxide (pCO_2_)	39	44	41
Bicarbonate	24	20	21
Arterial base excess	-0.8	-5.8	-3.6

With a high index of suspicion for thyrotoxicosis, the serum T3, free T4 and TSH concentrations were measured and revealed a significantly elevated T3 and free T4 along with suppressed TSH (Table 2). In light of her clinical manifestations, laboratory findings and precipitating factors (e.g., major surgery and recent exposure to intravenous iodinated contrast), a diagnosis of thyroid storm was made (with a Burch-Wartofsky score of 65 points). Her thyroid stimulating immunoglobulin (TSI) activity was at 217% of basal level and her TSH receptor antibody titer was 6.32 (normal, ≤1.75). Both values were consistent with Graves’ disease.

**Table 2:** Postoperative clinical and laboratory findings of hyperthyroid state.

Date	Day of surgery	Postoperative day 0	Postoperative day 1	Postoperative day 2	Postoperative day 4	Postoperative day 5	Postoperative day 8
Thyroid Stimulating Hormone (TSH)				0.02			
T3			485.0				
Free T4			>6.99		3.88	2.75	1.6

She was initially treated with propylthiouracil, an esmolol drip and saturated solution of potassium iodide (SSKI). Intravenous hydrocortisone was also administered. Her clinical status gradually improved. She was successfully extubated on postoperative day 3. Her vital signs normalized by postoperative day 6. On postoperative day 8, the free T4 concentration returned to a normal range. On an outpatient follow-up one week later, she was doing well. Histopathologic analysis of the mediastinal mass was consistent with thymic hyperplasia.

## Discussion

Our report demonstrates a surprising cause of perioperative tachycardia and respiratory failure. Postoperative tachycardia, especially within the first four days of surgery, should always raise concern for bleeding, pulmonary embolism or myocardial ischemia. Although most episodes of postoperative tachycardia will not be associated with a major complication, an appropriate evaluation must be performed to rule out these potentially catastrophic etiologies. In a study of 377 patients who underwent ventral hernia repair, 32% experienced at least one episode of postoperative tachycardia [10]. Serious adverse events including infections, bleeding, venous thromboembolism and cardiac events accounted for 20% of these patients. In another study of 4,621 patients undergoing hip and knee arthroplasty, a maximum heart rate greater than 110 beats/min had an odds ratio of 9.4 for pulmonary embolism [11].

TS deserves inclusion in the differential diagnosis of perioperative tachycardia, especially when the patient has hyperpyrexia and respiratory failure [12]. A substantial elevation in the metabolic rate caused by TS is inevitably associated with increased tissue oxygen consumption and carbon dioxide production, leading to hypoxemia and hypercapnia despite apparent hyperventilation as observed in the present patient. The differential diagnoses of perioperative TS should include other causes of hyperdynamic circulation such as malignant hyperthermia, neuroleptic malignant syndrome and pheochromocytoma [13]. The etiology of perioperative TS is multifactorial. In most cases, it is difficult to say that surgical stress is the only trigger as anesthetic agents, use of iodinated contrast, infection and psychological stress can also precipitate TS [14].

Thyroid storm following non-thyroid surgeries has rarely been reported. We have identified 24 such cases in the literature from 1930 to 2018. This condition is more common in middle-age women. The onset of TS can range from intraoperatively to postoperative day 3. The most common presentation is tachycardia, hyperpyrexia, acute anxiety and restlessness.

One study in 1947 reported 36 (1.8%) instances of TS among 2,033 hyperthyroid patients admitted between 1921 and 1946 [15]. Twenty-five cases of TS occurred following 1,383 surgeries performed on these patients. The authors referred to these instances as ‘surgical’ TS because all followed thyroidectomy, except for one hyperthyroid patient who underwent an urgent operation for lysis of peritoneal adhesions causing acute small bowel obstruction. In approximately half of the surgical patients, wound infection, pneumonia and postoperative hemorrhage were cited as precipitating factors for postoperative TS. In another series of 2,329 hospitalized hyperthyroid patients between 1949 and 1968, only 21 (0.9%) cases of TS were reported [16]. An apparent precipitating factor was identified in 13 (62%) patients with this adverse event. Surgical stress precipitated TS in two patients only; one of them undergoing thyroidectomy for Graves’ disease and the other a non-thyroid surgery. Another study in 1958 reported five instances of postoperative thyroid storm among 16,450 surgeries performed over a period of 12 years [17]. In a more recent Japanese nationwide survey, non-thyroid surgeries were reported as the trigger in eight (2.2%) of 356 patients with TS studied between 2004 and 2008 [18]. No instances of TS were caused by thyroid surgery, and the authors attributed this observation to improvement in the preoperative management of patients with Graves’ disease.

The aforementioned data suggest a shift in precipitating causes of TS over time. While thyroidectomy in patients with a previously diagnosed hyperthyroid state (Graves’ disease or toxic nodular goiter) was the most common trigger in the first half of the last century, non-surgical triggers dominated in the second half of the century owing to widespread use of modern antithyroid medications for preoperative preparation of thyrotoxic patients. In modern times surgical stressors, including thyroidectomy, incite only a small fraction of cases of TS.

Our patient had Graves’ disease and an anterior mediastinal mass that was ultimately shown to be thymic hyperplasia. The association between thymic hyperplasia and Graves’ disease was first described over a century ago by Matti and Halsted. The thymus and thyroid glands have common embryologic origins, as they arise from the third and fourth endodermal pharyngeal pouches, respectively. Recently, there has been a significant review of this benign association and its pathophysiological basis [19]. It remains unclear whether Graves’ disease causes thymic hyperplasia or results from it. Thyroid hormones exert a trophic effect on thymic epithelial cells and cortical lymphoid tissue. The thymus, on the other hand, possesses functional TSH receptors. As such, anti-TSH receptor antibodies present in Graves’ disease may directly activate thymic cells via these receptors. The association between thymic hyperplasia and Graves’ disease is usually unrecognized by physicians. In our patient, this association was appreciated retrospectively due to the missed preoperative diagnosis of Graves’ disease.

Our case study illustrates the importance of a proper preoperative assessment. The signs and symptoms of hyperthyroidism can be very subtle to clinically overt. Obtaining a detailed history from a patient with multiple comorbidities can be challenging at times. Family members are a crucial and useful source of clinical information in this setting or if subtle clinical changes are otherwise unnoticeable. Our patient had multiple comorbidities, was being treated for autoimmune conditions over a decade and had been seen by multiple clinicians across different specialties. Her TSH concentrations were low normal in two prior occasions measured years before the surgery. But no additional workup was conducted to rule out primary thyroid diseases. She had an unintentional weight loss in the past year associated with heat intolerance and bulging of the eyes. The preoperative assessment failed to recognize these features, and this important clinical information was only corroborated in an interview with patient’s relatives after surgery. Furthermore, the patient herself claimed her weight loss to be intentional. It is possible that, if the diagnosis of hyperthyroidism had been made preoperatively, the surgery may not have been necessary. Hyperthyroidism can stimulate thymic hyperplasia, and that may have been the causative factor for the hyperplasia in our patient.

## Conclusions

Hyperthyroidism may present with subtle, nonspecific and constitutional symptoms. A preoperative measurement of TSH concentration should be sought in patients with tachycardia, palpitation, labile blood pressure, unexplained weight changes or poorly controlled anxiety. The importance of proper preoperative history taking and physical exam cannot be overestimated. This adverse event could have potentially been avoided by identification of hyperthyroidism preoperatively through a more accurate exam and assessment. This entails interviewing available relatives in addition to the patient if longstanding comorbidities or multitude of misleading symptoms and signs are present. Although uncommon, TS should be considered in differential diagnosis of perioperative tachycardia and respiratory failure. Nonthyroid surgical stressors are more frequently being reported as the trigger of perioperative TS in the setting of poorly controlled or overlooked hyperthyroidism. Management of TS demands prompt diagnosis and aggressive therapy to ensure an uncomplicated recovery. The benign association between thymic hyperplasia and Graves’ disease merits attention in clinical practice.
